# Replication fork blocking deficiency leads to a reduction of rDNA copy number in budding yeast

**DOI:** 10.1016/j.isci.2024.109120

**Published:** 2024-02-06

**Authors:** Taichi Murai, Shuichi Yanagi, Yutaro Hori, Takehiko Kobayashi

**Affiliations:** 1 Laboratory of Genome Regeneration, Institute for Quantitative Biosciences (IQB), The University of Tokyo, 1-1-1 Yayoi, Bunkyo-ku, Tokyo 113-0032, Japan; 2 Department of Biological Sciences, Graduate School of Science, University of Tokyo, 7-3-1 Hongo, Bunkyo-ku, Tokyo 113-0033, Japan; 3 Collaborative Research Institute for Innovative Microbiology**,** The University of Tokyo, 1-1-1 Yayoi, Bunkyo-ku, Tokyo 113-0032, Japan

**Keywords:** Genetics, Molecular biology, Cell biology

## Abstract

The ribosomal RNA genes are encoded as hundreds of tandem repeats, known as the rDNA, in eukaryotes. Maintaining these copies seems to be necessary, but copy number changes in an active manner have been reported in only frogs, flies, *Neurospora*, and yeast. In the best-studied system, yeast, a protein (Fob1) binds to the rDNA and unidirectionally blocks the replication fork. This block stimulates rDNA double-strand breaks (DSBs) leading to recombination and copy number change. To date, copy number maintenance and concerted evolution mediated by rDNA repeat turnover were the proposed benefits of Fob1-dependent replication fork arrest. In this study, we tested whether Fob1 provides these benefits and found that rDNA copy number decreases when *FOB1* is deleted, suggesting that Fob1 is important for recovery from low copy number. We suppose that replication fork stalling at rDNA is necessary for recovering from rDNA copy number loss in other species as well.

## Highlights

• In the absence of Fob1, rDNA (rRNA gene) copy number decreases along with the generations
• The copy number of rDNA has little effect on growth, except for extremely low copies
• rDNA copy number is regulated according to the total copy in a cell not for each locus

## Graphical abstract

**Figure:**
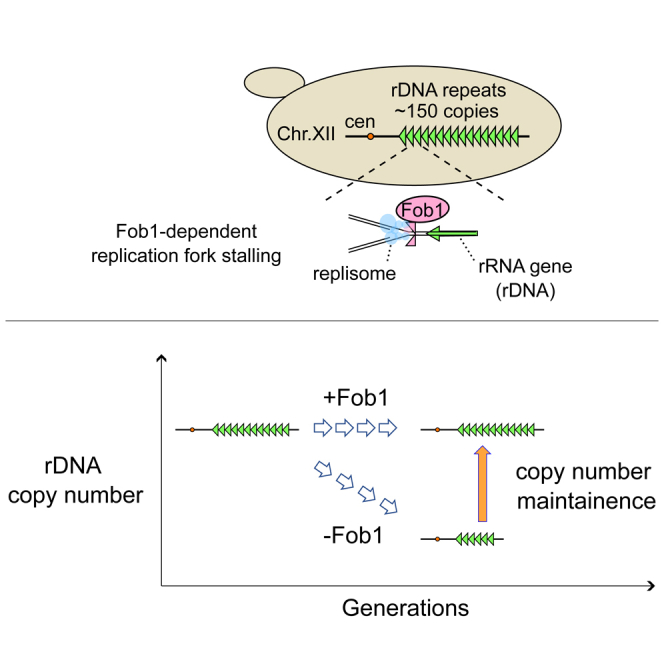

## Introduction

The ribosomal RNA gene (rDNA) loci, from which rRNA is transcribed, are arranged as tandem repeats of rDNA units in most eukaryote genomes. While it is often omitted in reference sequences, organisms typically carry tens or hundreds of rDNA units, varying between species (e.g., budding yeast,^1^ nematode [*Caenorhabditis elegans*],^2^ fruit fly [*Drosophila melanogaster*],^3^ frog [*Xenopus laevis*],^4^ mouse, and human.^5^). Although multiple copies of rDNA might be necessary to produce adequate numbers of ribosomes—for example, ∼2,000 ribosomes are produced every minute in budding yeast^6^—a substantial number of copies are transcriptionally completely silent even in rapidly growing cells.^7^^,^^8^^,^^9^ This suggests that rDNA copies are in excess.

**Figure 1:**
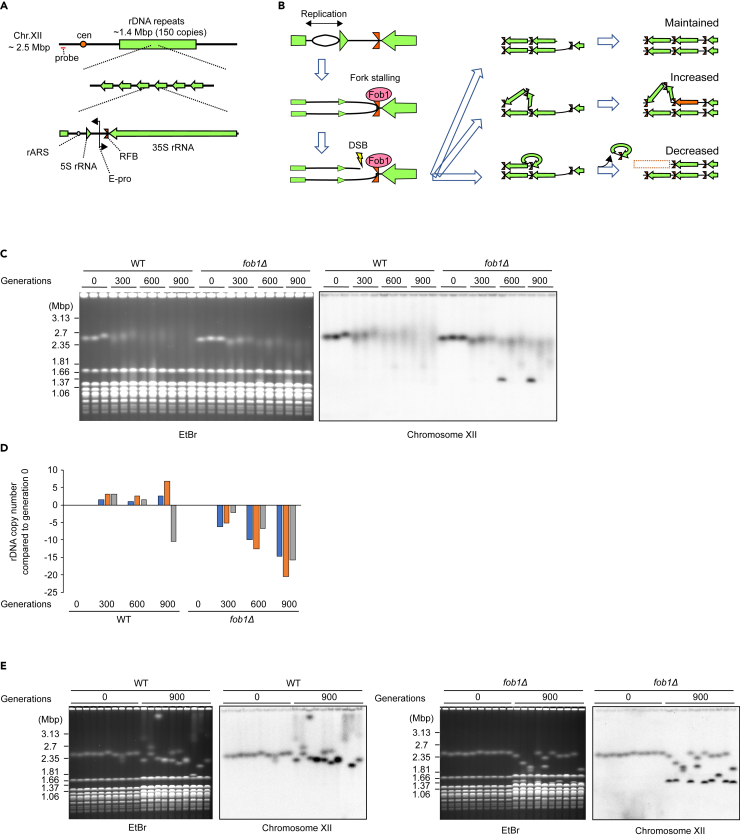
rDNA copy number decreases in a *fob1Δ* strain over time (A) Schematic of yeast rDNA locus. The yeast rDNA locates on chromosome XII, and contains 35S and 5S rRNA genes, an origin of replication (rARS), a non-coding RNA promoter (E-pro), and a replication fork barrier (RFB) site where Fob1 binds. Red bar indicates the position of the probe used for southern hybridization. (B) Fob1-dependent DSB formation and rDNA recombination. Replication fork stalling occurs when Fob1 binds to RFB, and DSBs occur in a proportion of the stalled forks. Copy number variation emerges through unequal sister chromatid recombination on repairing the DSBs. (C) Genomic DNAs extracted from WT and *fob1Δ* strains following growth for the number of generations indicated were separated by PFGE. Chromosome XII size was monitored by ethidium bromide staining and by a radioactive probe specific to the left arm of chromosome XII. (D) Shift of rDNA copy number relative to generation 0 was calculated from the signal peak densities of chromosome XII and the *H. wingei* markers in the ethidium bromide-stained gel image from (C). (E) Single colonies were isolated from the 0 and 900 generation cultures for each strain (lane 1, 10, 13, and 22 in C), and their genomic DNAs were monitored as in (C).

Excess rDNA copies imply cells have systems to maintain copy number above the minimally required level for survival. Consistent with this, recovery of rDNA copy number from decreased states has been reported in oocytes of frog,^4^ germline stem cells (GSCs) of fruit fly,^10^ *Neurospora*,^11^ and budding yeast, *Saccharomyces cerevisiae*.^12^ In budding yeast, additionally to 35S rRNA gene, which corresponds to mammalian 47S rRNA gene, each unit of rDNA repeats harbors several elements between the genes (Figure 1A). Among those, recovery of copy number from deletions depends on replication fork barrier (RFB) site and Fob1, which binds to RFB and stalls replication forks that move toward the 35S rRNA transcription direction.^13^^,^^14^ Fob1-mediated fork stalling results in DNA double-strand breaks (DSBs) at the RFB,^15^^,^^16^^,^^17^^,^^18^ with copy number recovery being a consequence of unequal recombination between sister chromatids during repair of these DSBs (Figures 1A and 1B). In mutants lacking Fob1, replication fork stalling and DSBs at the RFB are abolished, and copy number recovery is suppressed.^12^^,^^13^ This Fob1-dependent rDNA copy number variation in yeast is linked to a mechanism of rDNA copy number regulation mediated by Sir2 and UAF (upstream activating factor).^19^^,^^20^ Sir2 is a histone deacetylase that stabilizes rDNA copy number by suppressing rDNA noncoding transcription,^21^^,^^22^ and its expression is regulated by UAF in an rDNA copy number-dependent manner. When rDNA copies are in the wild-type (WT) range, UAF, which binds to the 35S rRNA promoter and enhances rRNA transcription, is titrated by this rDNA association and transcription of Sir2 stabilizes rDNA copy number. Conversely, when rDNA copies are low, the excess UAF which cannot bind to rDNA instead binds to the promoter region of the *SIR2* gene and suppresses Sir2 transcription.^20^ This stimulates Fob1-dependent recombination, leading to rDNA copy number amplification and allowing yeast to recover the rDNA copy number back to the wild-type level.

Another consequence of Fob1-mediated rDNA recombination is homogenization of the rDNA. The multi-copies of the rDNA within a genome maintain high levels of sequence identity, even though the sequence can change between species.^23^ This unusual evolutionary pattern, which is termed concerted evolution, is believed to result from recombination between the rDNA units resulting in copy number change and thus continual turnover of rDNA repeat units. A consequence of this turnover is that all repeats in a genome are recently descended from a single ancestral repeat, and so have limited sequence divergence. This turnover process resulting in concerted evolution is known as homogenization, and in budding yeast, the dependence of rDNA recombination on Fob1 suggests that Fob1 is a necessary factor for rDNA homogenization.

The apparent roles of Fob1 in rDNA copy number recovery and homogenization suggest that it is important for maintenance of many functional rDNA copies. Interestingly, however, *FOB1* only shows limited conservation, being found in the family Saccharomycetaceae but apparently not beyond. Nevertheless, analogous fork arrest exists in other organisms (e.g., REB in pombe,^24^ mouse,^25^ and human^26^), although to date copy number regulation in an active manner has only been reported in frogs,^4^ fruit flies,^10^ *Neurospora*,^11^ and yeast.^12^ Additionally, while *FOB1* is required for efficient recovery from rDNA copy number loss, deletion of *FOB1* stabilizes rDNA copy number and so suppresses the copy number loss itself. Moreover, recombination in rDNA is associated with detrimental phenotypes. In yeast, increased copy number variation shows reduced replicative lifespan,^27^^,^^28^^,^^29^ and accumulation of extrachromosomal rDNA circles (ERCs) produced by Fob1-dependent recombination may be a cause.^30^^,^^31^^,^^32^ Furthermore, in human, patients with Bloom syndrome, a genetic disorder associated with compromised genomic repair, show increased rDNA copy number variation,^33^^,^^34^ and structural alterations to the rDNA cluster were observed in a subset of adult solid tumor samples.^35^ Thus, rDNA recombination presents an evolutionary conundrum, as it not be necessary or could be even harmful, yet its presence across a number of species suggests there must be some advantage to its existence. Here, to investigate the roles of replication fork blocking activity in rDNA copy number maintenance and homogenization, we passaged wild type and *FOB1*-deleted strains for hundreds of generations to assess the long-term impact of rDNA recombination defective condition.

## Results

### rDNA copy number decreases in *fob1Δ* mutant

To examine the role of Fob1-mediated recombination, we performed a long-term evolution experiment in wild type and *FOB1*-deleted (*fob1Δ*) strains by subjecting strains to serial 1,000-fold dilution transfers (approximately 10 generations per transfer) for a total of ∼900 generations. Previous studies showed that deletion of *FOB1* stabilizes rDNA copy number in yeast by suppressing copy number variation of rDNA by recombination,^12^^,^^31^^,^^32^ therefore, we first assessed whether this was the case in our experiment. Genomic DNAs of the long-term cultures were separated by pulsed field gel electrophoresis (PFGE) and rDNA copy number was assessed from the size of the chromosome XII band. As expected, comparison between strains of the same generations shows that the wild-type chromosome XII bands are more smeared than those of *fob1Δ* (Figure 1C), indicating greater copy number variation. Nevertheless, the chromosome XII bands did become smeared in *fob1Δ* strains as generations increased, suggesting that copy number variation does still occur in *fob1Δ*. Strikingly, however, while the 900 generation chromosome XII bands were smeared in both directions in the wild-type replicates, those of the *fob1Δ* replicates were mostly smeared downwards in copy number (Figure 1C). To quantify this, we estimated the rDNA copy number from the size of chromosome XII peak intensity over the course of the long-term culture. This showed no particular direction of copy number change over the experiment in wildtype, consistent with stochastic copy number variation, but for *fob1Δ* the chromosome XII peak intensity monotonically shifted toward fewer copy numbers (Figure 1D). Additionally, the amount of ERCs monotonically increased in *fob1Δ* along with generations (Figures S1A and S1B). We suppose the increasing amount of ERCs may be compensating the loss of genomic rDNA. In fact, in wild-type strains, cells with low-copy genomic rDNA retain more ERCs, presumably to compensate the number of total rDNA units.^36^

To confirm the phenomenon of copy number reduction in long-cultured *fob1Δ* strains, single colonies were isolated from the 900-generation strains, and the lengths of their chromosome XII were assessed by PFGE. Consistent with the bulk population results, chromosome XII size had contracted in most *fob1Δ* colonies, while expansions were observed more frequently in wild-type colonies (Figure 1E). These results suggest that rDNA copy number gradually decreases when Fob1 is absent.

**Figure 2:**
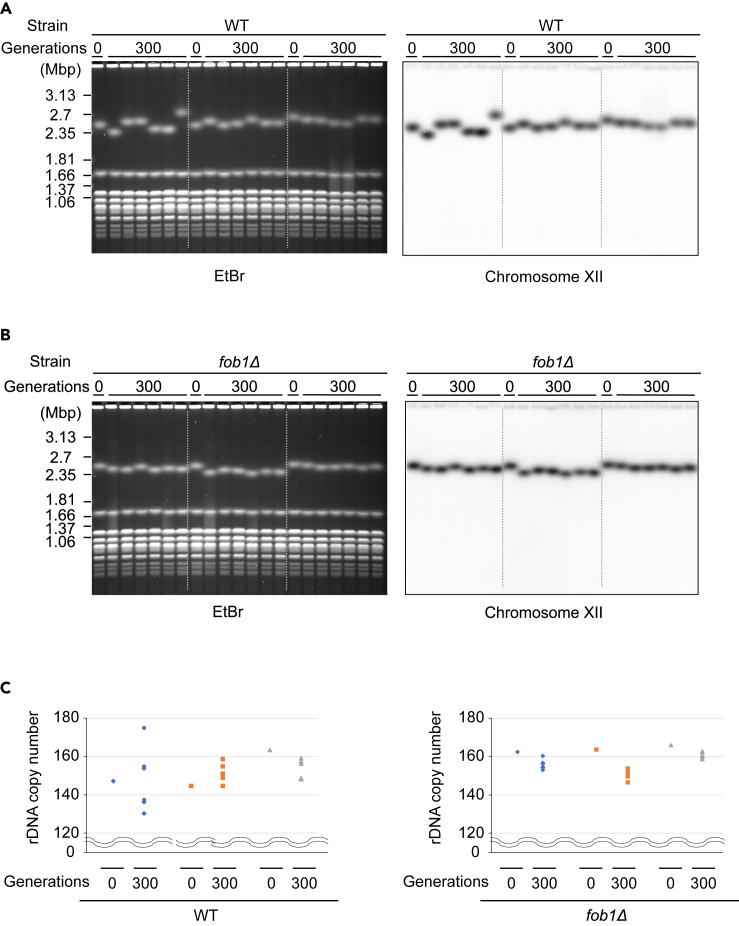
rDNA copy number shift in haploid cells (A and B) Haploid cells were passaged by single-colony transfer on plates and genomic DNA from the wild type (A) and *fob1Δ* (B) strains was monitored as in Figure 1C. Three individual colonies from generation 0 and six colonies from generation 300 derived from each generation 0 isolate are shown. Lanes for each isolate are separated by dotted lines. (C) The rDNA copy number of each colony from (A) and (B) was estimated as in Figure 1D.

Unexpectedly, we observed two bands of chromosome XII in most colonies isolated from generation 900 (Figures 1E, S1C, and S1D). Especially in *fob1Δ,* some of those bands were relatively small and overlapped with other chromosomes in the ethidium bromide-stained gel. Although we initiated this long-term culture with haploid strains, these double bands could be a consequence of diploidization, as has been observed previously in *S. cerevisiae* long-term evolution experiments,^37^ leading to domination by diploid cells. In fact, we confirmed diploidization of these cultures by showing that the DNA content had doubled from the original strains (Figure S2). Moreover, to verify that reduction in rDNA copy number in *fob1Δ* occurs regardless of ploidy, we performed another long-term culture experiment where wild type and *fob1Δ* haploids were cultured solely by single-colony transfers on plates. PFGE assessment of chromosome XII sizes of single colonies from these strains following ∼300 generations of growth again showed both expanded and contracted rDNA copy numbers in wild type but only contracted copy numbers in *fob1Δ* (Figure 2), although the level of copy number reduction was not as great as in the 900-generation colonies. Thus, the rDNA shrinking phenotype in *fob1Δ* is observed regardless of ploidy.

### Very low copy number affects growth rate

**Figure 3:**
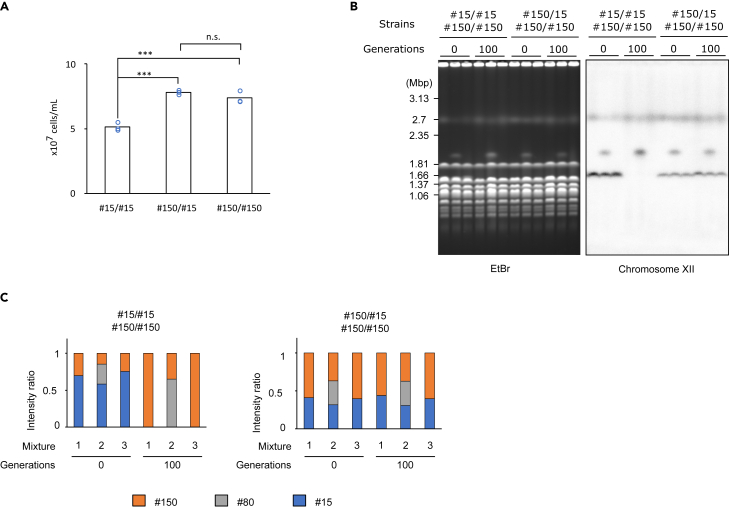
Low rDNA copy number reduces growth rate (A) Saturated liquid cultures were diluted by 1,000-fold, and cell densities were measured after 12 h. Bars show the average for the 3 replicates (shown as circles), and error bars show ± SEM. ∗∗∗: p < 0.001, n.s.: p > 0.05, Tukey’s test. (B) Cells with different rDNA copy numbers (#N = copy number) were mixed at equal cell numbers and passaged for 100 generations. The genomic DNAs were monitored as in Figure 1C. (C) The signal intensities of chromosome XII with different rDNA copy numbers from (B) were measured, and the proportions of signal intensities for each lane are plotted.

Contraction in rDNA copy number in *fob1*Δ strains could either be a consequence of Fob1-independent recombination resulting primarily in loss of copies, or as a result of natural selection if a smaller rDNA copy number is beneficial in a *fob1Δ* background (Kobayashi, 2008). To test the latter possibility, we compared the growth of *fob1Δ* with different rDNA copy numbers. Stationary-phase liquid cultures were diluted 1,000-fold and cell density was measured after 12 h of growth. Comparing growth among diploid strains showed that strains carrying 15 rDNA copies in both chromosomes (RDN1#15/RDN1#15) proliferated slower than those carrying higher rDNA copy numbers (Figure 3A). Additionally, enhanced growth was observed in both wild type and *fob1Δ* strains during the long-term culture (Figure S3), as is often observed in continuous culture studies.^38^^,^^39^^,^^40^ The comparative growth of wild type and *fob1Δ* suggests that the enhanced growth is independent of *FOB1* or rDNA. As an independent test of whether smaller rDNA copy number is beneficial, strains with different rDNA copy numbers were mixed at the same cell density and competed for ∼100 generations. The proportion of each strain was then estimated by comparing the band intensity of chromosome XII. In competitions between RDN1#15/RDN1#15 and RDN1#150/RDN1#150 (where #N indicates copy number) strains, the 15-copy rDNA chromosome XII band completely disappeared, indicating extinction of the low copy strain (Figures 3B and 3C). However, the proportion of chromosome XII band sizes was almost unchanged in the RDN1#150/RDN1#15 and RDN1#150/RDN1#150 strain competition (Figures 3B and 3C). In one RDN1#150/RDN1#150 replicate there was an unexpected chromosome XII band, representing about 80 rDNA copies, which is presumably a consequence of stochastic loss of rDNA copies in this replicate. Nevertheless, the RDN1#150/RDN1#80 replicate still outcompeted the RDN1#15/RDN1#15 strain, further corroborating the low competitiveness of strains with 15 copies. Additionally, unexpected ploidy change was not observed in these strains (Figure S4), indicating the difference in fitness caused purely by rDNA copy number. Thus, we find that highly reduced rDNA copy number negatively affects growth, while intermediate rDNA copy number has little discernable impact on growth. These results indicate that the *fob1Δ* rDNA shrinking phenotype is not a consequence of selection for low rDNA copy numbers, and thus is likely to instead result from a bias of Fob1-independent recombination toward deletion of rDNA copies.

### Copy number variation is observed for both chromosomes in diploids

**Figure 4:**
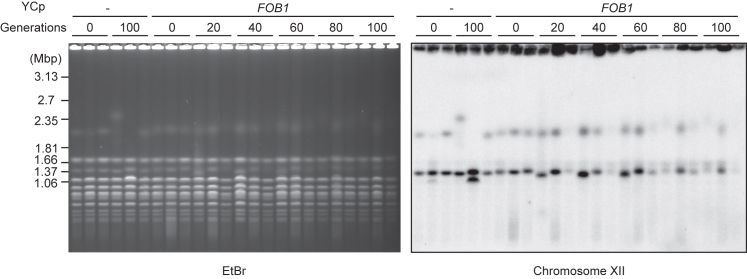
Copy number recovery in diploid cells operates on total copy number A diploid strain (*fob1Δ*/*fob1Δ*) was transformed with a single-copy plasmid (YCp) with (*FOB1*) or without (−) the *FOB1* gene and cultured for the indicated number of generations. Genomic DNA was monitored as in Figure 1C.

Recovery from low copy rDNA numbers is a characterized function of Fob1,^12^ but it is unclear whether this copy number recovery system works on total copy number or on each chromosome independently in diploids. To investigate this, we transformed a single-copy plasmid with or without the *FOB1* gene into the 900-generation *fob1Δ* strain (colony 1, YTM511) and monitored chromosome XII size by PFGE following 100 generations. If rDNA copy numbers on homologous chromosomes are controlled independently, the chromosome with lower rDNA copies should show a greater copy number increase, which would manifest as the smaller chromosome XII being more smeared than the larger one in the PFGE analysis. In contrast, however, we found that the larger chromosome became more smeared than the smaller one (Figure 4). These results indicate that diploid cells recover rDNA copy number based on total copy number rather than the copy number of each chromosome.

### Deletion of Fob1 did not result in an increase in rDNA mutations in the current experimental period

The reduced smearing of the chromosome XII band indicates that rDNA recombination frequency is significantly lower in *fob1Δ*, thus we wondered whether *fob1Δ* may carry a higher burden of rDNA mutations as a result of impaired homogenization. To investigate this, we first performed base-level mutation analysis using Illumina short-read sequencing by PCR amplifying and sequencing the rDNA, and analyzing the resultant sequences. However, the frequency of variants from the reference sequence was comparable between WT and *fob1Δ* (Figure S5). This suggests that the accumulation of point mutations in the *fob1Δ* strain is not greater than the accumulation in wild type, at least in the context of the background mutations in the rDNA and the rate introduced by PCR and sequencing.

Next, to investigate larger genomic mutations, we utilized Oxford Nanopore sequencing. Due to the higher rate of sequencing errors in Oxford Nanopore sequencing, we focused on deletion mutations larger than 50 nt as they are more easily discernible, while insertion mutations are sometimes difficult to distinguish from sequencing errors. Similar to the Illumina point mutation results, we did not observe significant differences in the rate of deletion mutations between wild type and *fob1Δ* strains. Our findings suggest that Fob1 does not function to reduce the yeast rDNA mutational burden, at least over the time course of this experiment.

## Discussion

Here, we found that Fob1, which accelerates rDNA instability by enhancement of recombination and shortens replicative lifespan, is required to maintain rDNA copy number (Figures 1, 2, S1C, and S1D). This also suggests repetitive genes like rDNA lose copies if there is no strong selective pressure. We show that cells lose fitness once the copy number becomes too low, so the ability to recover copies is truly beneficial (Figure 3). Furthermore, Fob1’s ability to rectify rDNA copy number may also be beneficial for maintaining additional “non-transcribed copies” above those strictly required for ribosome production, given that low-copy rDNA strains are more vulnerable to external stresses such as DNA damage caused by UV and MMS.^41^ Moreover, we observed a steady increase in the amount of ERC in *fob1Δ* along with the generations (Figures S1A and S1B). This might be compensating growth rate in the long-cultured *fob1Δ,* which has less genomic rDNA.^36^ However, ERC accumulation in the mother cell would shorten replicative lifespan, further implying disadvantages of losing *FOB1* gene in a long-term perspective. Our results also show that Fob1-mediated rectification of rDNA copy number is regulated based on the total copy number in a cell, rather than the copy number on each homologous chromosome (Figure 4). These results are consistent with the recently described rDNA copy number counting and recovery mechanism mediated by Sir2 and UAF,^20^ as this mechanism is based on total copy number rather than on chromosome-specific copy number.

Defects caused by insufficient rDNA copies are also reported in flies and worms. In flies, *Drosophila melanogaster*, shortage of rDNA copies causes bobbed phenotype, which is indicative for short bristles and slow growth.^42^ Also, in worms, *Caenorhabditis elegans*, the more they lose rDNA copies, the severer their phenotype becomes.^43^ The threshold for low copy may differ between organisms, but in both flies and worms, some progenies from copy number-reduced parents show increased rDNA copies, implying copy number recovery in these organisms.^10^^,^^43^ In fact, in flies, a protein for efficient copy number recovery was identified,^44^ and further research is awaited.

In contrast to the evidence for the role of Fob1 in copy number maintenance, we found no evidence for Fob1 playing a role in reducing rDNA repeat mutation accumulation (Figure S5). The lack of difference in the mutation rate between wild type and *fob1Δ* was unexpected. High rates of transcription are linked to elevated mutation rates,^45^ thus the highly transcribed rDNA is expected to experience a relatively high mutation rate. Moreover, the documented role of Fob1 in promoting rDNA copy turnover by recombination is expected to be a homogenizing force,^23^^,^^46^ thus more mutations were expected to be retained in *fob1Δ*. The reason we did not see an accumulation of mutations in *fob1Δ* could be because the mutation rate is too low to detect a difference over the number of generations we assayed,^47^ particularly because background mutations weaken our ability to detect small differences. This could be rectified by using mutator strains in which DNA repair activity is reduced, elevating the mutation rate.^48^^,^^49^ Alternatively, single colonies could be isolated and sequenced, as currently the sequencing is done on population of cells, which makes it difficult to detect mutations that have occurred in a single rDNA repeat in a single cell. It is possible that much greater timescale of culture, such as over 9,000 generations, proves Fob1-mediated homogenization of the rDNA.

While expansions of rDNA were largely suppressed in *fob1Δ,* we did observe contractions of chromosome XII. The fact that we did not detect an increased rate of deletions in *fob1Δ* through the Nanopore sequencing indicates that the contractions in chromosome XII size are caused by mechanisms utilizing homology, such as homologous recombination after looping or single-strand annealing (SSA), rather than a mechanism such as non-homologous end joining that result in internal rDNA deletions (Figures S6A and S6B). Nevertheless, we did observe some colonies with expanded chromosome XII in diploids in *fob1Δ* (Figure S1C lane 6, S1D lane 4, 7, 12, 15, 23, and 24). As such chromosome XII expansions were not found in *fob1Δ* haploids (Figure 2) and some *fob1Δ* diploids with an expanded chromosome XII also had a significantly contracted chromosome XII (Figure S1D lane 12 and 24), we hypothesize that unequal recombination occurred between homologous chromosomes, rather than between sister chromatids which is also possible in haploids (Figure S6C). On the chromosome XII in wild type, recombination with the homologous chromosome occurs more frequently than in *fob1Δ*,^50^ but Fob1-dependent sister chromatid recombination is dominant for copy number recovery.^12^

In this study, we confirmed that, in yeast, Fob1 is necessary to maintain and recover rDNA copy number, presumably through its replication fork stalling activity. We think it is likely that fork stalling is necessary to maintain rDNA copy number in other organisms as well, but studying the relationship between replication fork stalling and rDNA copy number is challenging due to the multiple functions of fork stalling proteins in other systems. In humans, for instance, TTF1 (transcription termination factor 1), which is necessary for fork stalling at rDNA, also plays a crucial role in efficient transcription of rRNA, making it indispensable. In yeast, Fob1 is responsible for fork stalling, while Reb1 and Nsi1 are necessary for transcription termination and have little effect on fork stalling. Thus, yeast has assigned rRNA transcription termination and replication fork stalling to distinct proteins, but why this different approach has evolved is not known. Further investigations in other organisms are needed to elucidate the general significance of rDNA replication fork stalling.

### Limitations of the study

Here, we showed that rDNA copy number decreases in the absence of Fob1, but we have not elucidated the molecular mechanism causing this phenotype. Also, from the perspective of evolution, ∼900 divisions are too few, and the laboratory condition during passage was far from the wild. In a harsher condition, more mutations might accumulate in the rDNA, and additional defects would be observed in *fob1Δ.*

## STAR★Methods

### Key resources table

**Table:** 

REAGENT or RESOURCE	SOURCE	IDENTIFIER
**Chemicals, peptides, and recombinant proteins**		

RNase A	Macherey-Nagel	Cat# 740505
2-mercaptoethanol	Wako	Cat# 131-14572
Proteinase K	Nacalai	Cat# 29442-85
Zymolyase 100T	Nacalai	Cat# 07665-55
SeaPlaque GTG Agarose	Lonza	Cat# 50111
Pulsed Field Certified Agarose	BIO-RAD	Cat# 1620138
Hybond N+	GE Healthcare	Cat# RPN203B
STAR agarose	RIKAKEN	Cat# RSV-AGRP

**Critical commercial assays**		

Random Primer DNA Labeling Kit Ver.2	Takara	Cat# 6045
ProbeQuant G-50 Micro Columns	GE Healthcare	Cat# 28903408
Flongle Flow Cell	Oxford Nanopore	Cat# FLO-FLG001
Ligation Sequencing Kit	Oxford Nanopore	Cat# SQK-LSK109

**Deposited data**		

Whole genome sequencing data	Illumina	SRA: PRJNA1009244
Long read whole genome sequencing data	Oxford Nanopore	SRA: PRJNA1009244
Raw data for electrophoresis, flow cytometry, and growth rate.	Mendeley	https://data.mendeley.com/datasets/vxw3zvb6m9/draft?a=cc2f8aa0-139c-4f12-8e0f-9e9ea227d733

**Experimental models: Organisms/strains**		

S. cerevisiae, BY4741 strain: YTM33, *MATa his3Δ1 leu2Δ0 met15Δ0 ura3Δ0*	ATCC	ATCC: 201388
S. cerevisiae, BY4741 strain: YTM224, *MATa his3Δ1 leu2Δ0 met15Δ0 ura3Δ0 fob1Δ::NatMX*	This paper	N/A
S. cerevisiae, BY4741 strain: YTM446-YTM448, *MATa his3Δ1 leu2Δ0 met15Δ0 ura3Δ0* 0 generation	This paper	N/A
S. cerevisiae, BY4741 strain: YTM453, *MATa his3Δ1 leu2Δ0 met15Δ0 ura3Δ0 fob1Δ::NatMX* 0 generation	This paper	N/A
S. cerevisiae, BY4741 strain: YTM486-YTM488, *MATa his3Δ1 leu2Δ0 met15Δ0 ura3Δ0* 300 generations	This paper	N/A
S. cerevisiae, BY4741 strain: YTM489-YTM491, *MATa his3Δ1 leu2Δ0 met15Δ0 ura3Δ0 fob1Δ::NatMX* 300 generations	This paper	N/A
S. cerevisiae, BY4741 strain: YTM498-YTM500, *MATa his3Δ1 leu2Δ0 met15Δ0 ura3Δ0* 600 generations	This paper	N/A
S. cerevisiae, BY4741 strain: YTM501-YTM503, *MATa his3Δ1 leu2Δ0 met15Δ0 ura3Δ0 fob1Δ::NatMX* 600 generation	This paper	N/A
S. cerevisiae, BY4741 strain: YTM510-YTM512, *MATa his3Δ1 leu2Δ0 met15Δ0 ura3Δ0 900* generation	This paper	N/A
S. cerevisiae, BY4741 strain: YTM513-YTM515, *MATa his3Δ1 leu2Δ0 met15Δ0 ura3Δ0 fob1Δ::NatMX* 900 generation	This paper	N/A
S. cerevisiae, BY4741 strain: YTM618, *MATa his3Δ1 leu2Δ0 met15Δ0 ura3Δ0 fob1Δ::NatMX* single colony isolated from YTM513	This paper	N/A
S. cerevisiae, BY4741 strain: YTM647-YTM649, *MATa his3Δ1 leu2Δ0 met15Δ0 ura3Δ0 fob1Δ::NatMX YCplac33* 0 generation	This paper	N/A
S. cerevisiae, BY4741 strain: YTM687-YTM689, *MATa his3Δ1 leu2Δ0 met15Δ0 ura3Δ0 fob1Δ::NatMX YCplac33* 100 generation	This paper	N/A
S. cerevisiae, BY4741 strain: YTM651-YTM653, *MATa his3Δ1 leu2Δ0 met15Δ0 ura3Δ0 fob1Δ::NatMX YCplac33-FOB1p-FOB1* 0 generation	This paper	N/A
S. cerevisiae, BY4741 strain: YTM659-YTM661, *MATa his3Δ1 leu2Δ0 met15Δ0 ura3Δ0 fob1Δ::NatMX YCplac33-FOB1p-FOB1* 20 generation	This paper	N/A
S. cerevisiae, BY4741 strain: YTM667-YTM669, *MATa his3Δ1 leu2Δ0 met15Δ0 ura3Δ0 fob1Δ::NatMX YCplac33-FOB1p-FOB1* 40 generation	This paper	N/A
S. cerevisiae, BY4741 strain: YTM675-YTM677, *MATa his3Δ1 leu2Δ0 met15Δ0 ura3Δ0 fob1Δ::NatMX YCplac33-FOB1p-FOB1* 60 generation	This paper	N/A
S. cerevisiae, BY4741 strain: YTM683-YTM685, *MATa his3Δ1 leu2Δ0 met15Δ0 ura3Δ0 fob1Δ::NatMX YCplac33-FOB1p-FOB1* 80 generation	This paper	N/A
S. cerevisiae, BY4741 strain: YTM691-YTM693, *MATa his3Δ1 leu2Δ0 met15Δ0 ura3Δ0 fob1Δ::NatMX YCplac33-FOB1p-FOB1* 100 generation	This paper	N/A
S. cerevisiae, BY4741 strain: YTM695-YTM697, *MATa his3Δ1 leu2Δ0 met15Δ0 ura3Δ0* 0 generation	This paper	N/A
S. cerevisiae, BY4741 strain: YTM698-YTM700, *MATa his3Δ1 leu2Δ0 met15Δ0 ura3Δ0 fob1Δ::NatMX* 0 generation	This paper	N/A
S. cerevisiae, BY4741 strain: YTM713-YTM715, *MATa his3Δ1 leu2Δ0 met15Δ0 ura3Δ0* 300 generation	This paper	N/A
S. cerevisiae, BY4741 strain: YTM716-YTM718, *MATa his3Δ1 leu2Δ0 met15Δ0 ura3Δ0 fob1Δ::NatMX* 300 generation	This paper	N/A

**Oligonucleotides**		

Primer for ERC probe, HS204: CATTTCCTATAGTTAACAGGACATGCC	Hosoyamada et al.^51^	N/A
Primer for ERC probe, HS205: AATTCGCACTATCCAGCTGCACTC	Hosoyamada et al.^51^	N/A
Primer for PFGE probe, HS210: GGCGAGGTTCAGAAAACTGTCG	Hosoyamada et al.^51^	N/A
Primer for PFGE probe, HS211: AAACGGCAAGAATGCGTTGTTTG	Hosoyamada et al.^51^	N/A
Primer for Illumina sequencing, oTM302: ATCATCATTCCCTAGAAACTGCC	This paper	N/A
Primer for Illumina sequencing, oTM303: GGCAAGTTCCAGAGAGGCAG	This paper	N/A

**Recombinant DNA**		

YCplac33	Gietz and Sugino^52^	ATCC: 87586
YCplac33-FOB1p-FOB1	Iida and Kobayashi^20^	N/A

**Software and algorithms**		

Multi Gauge	FUJIFILM	https://www.ualberta.ca/biological-sciences/media-library/mbsu/fla-5000/mulitgauge20.pdf
BWA (v0.7.17)	Li^53^	https://github.com/lh3/bwa
Trim Galore (v0.6.6)	Martin^54^	https://www.bioinformatics.babraham.ac.uk/projects/trim_galore/
Samtools (v1.10)	Li et al.^55^	https://www.htslib.org/
Naive Variant Caller	BlankenBerg et al.^56^	https://github.com/blankenberg/nvc
Python version 3.10.9	Python Software Foundation	https://www.python.org/
Guppy (v.6.1.5)	Oxford Nanopore	https://community.nanoporetech.com/

### Resource availability

#### Lead contact

Further information and request for resources and reagents should be directed to and will be fulfilled by the lead contact, Takehiko Kobayashi (tako2015@iqb.u-tokyo.ac.jp).

#### Materials availability

All materials generated in this study are available from the lead contact upon request.

#### Data and code availability

• Sequencing data and other raw data have been deposited at Sequence Read Archive (SRA) and Mendeley Data, respectively. Accession numbers are listed in the key resources table.
• This paper does not report original code.
• Any additional information required to reanalyze the data reported in this paper is available from the lead contact upon request.

### Experimental model and study participant details

#### Yeast strains, plasmids, primers and culture conditions

Yeast strains, plasmids and primers used in this study are listed in key resources table. Yeast cells were cultured at 30°C, and cells without plasmids were cultured in YPD media (10 g/L yeast extract, 20 g/L peptone and 20 g/L glucose), with 20 g/L Difco Bacto Agar added for solid medium. Plasmid-transformed strains were cultured in Synthetic Complete media (SC) without uracil. SC media used in this study was modified from Hartwell’ Complete media, as previously described.^57^ For the construction of *fob1Δ*, 100 μg/mL of clonNAT (Werner BioAgent) was added to YPD solid medium.

### Method details

#### Passage of yeast

For passages in liquid medium, single colonies were inoculated to 5 mL of YPD (Figures 1 and S1) or SC glucose without uracil (Figure 4), cultured at 30°C until saturation, and 5 μL of saturated culture was transferred to 5 mL of a fresh liquid medium for the next passage. Each passage was performed at least 20 h after inoculation into YPD and 2–3 days for SC. For the passages on plate medium, we first picked up three independent colonies from the wild-type and *fob1Δ* strains and streaked them onto YPD plates. After 2 days of incubation, a 1–2 mm colony was picked from each plate, and the number of cells in each colony was counted. The average cell number was 1.165 × 10^7^ (≈2^23.5^), therefore formation of a 1∼2 mm-sized colony corresponds to ∼20 generations. For each passage, six 1∼2 mm-sized colonies were picked up and mixed into 1mL of YPD, and 2 μL of them were steaked onto YPD plates for the next passage.

#### Competition assay

Single colonies of known rDNA copy number strains were inoculated into YPD liquid medium and cultured until saturation. To make cell mixture for 0 generation, cell density for each culture was measured, and cultures for strains with different rDNA copy number were mixed as the cell number for each strain becomes equal. 5 μL of the mixed culture were inoculated into 5 mL of YPD liquid medium and were cultured until saturation. From the second passage, cells were transferred as the usual passage.

#### Genomic DNA preparation in plugs

DNA plugs for Pulse-Field Gel Electrophoresis analysis were prepared using a previously described method (Yanagi et al., 2022) as follows. In this study, cells were inoculated directly from stock into liquid medium and incubated until saturation, except when a single colony was isolated. Cells from the saturated culture medium (5 × 10^7^ cells per plug) were collected and washed twice with 50 mM EDTA (pH 7.5). For each agarose plug, washed cells were resuspended in 33 μL of 50 mM EDTA (pH 7.5) and then mixed with 66 μL of solution I containing 8.3 mg/mL low-melting-point agarose SeaPlaque GTG (Lonza), 170 mM sorbitol, 17 mM sodium citrate, 10 mM EDTA (pH 7.5), 0.85% v/v b-mercaptoethanol, and 0.17 mg/mL Zymolyase 100 T (Nacalai). The solution was vortexed, poured into plug molds (Bio-Rad), and solidified at 4°C. Plugs were treated with a solution II containing 450 mM EDTA (pH 7.5), 10 mM Tris-HCl (pH 7.5), 7.5% v/v b-mercaptoethanol, and 10 mg/mL RNaseA (Macherey-Nagel) for 1 to 1.5 h at 37°C. Then, solution II was discarded and replaced with solution III containing 250 mM EDTA (pH 7.5), 10 mM Tris-HCl (pH 7.5), 10 g/L SDS and 1 mg/mL Proteinase K (Merck Millipore). The plugs were then incubated overnight at 50°C, washed four times with 50 mM EDTA (pH 7.5), and stored at 4°C.

#### Pulse field gel electrophoresis (PFGE) analysis

PFGE analysis was performed as previously described.^18^ Prepared plugs were cut to 3 mm width and DNA was separated using 1% agarose (Pulsed Field Certified Agarose, Bio-Rad) in 0.5× TBE on a Bio-Rad CHEF DR-III system with 2.4 L of 0.5× TBE and the following settings: 3.0 V/cm, run time = 68 h, included angle = 120°, initial switch time = 5 min, final switch time = 15 min, ramping factor = linear. *Hansenula wingei* chromosomal DNA markers (Bio-Rad) was used as a size marker.

#### ERC analysis

ERC analysis was performed as previously described.^51^ DNA plugs were cut to 5 mm width and DNA was separated using 0.4% agarose (STAR agarose, RIKAKEN) in 1× TAE on a Sub-cell GT electrophoresis system (Bio-Rad) in 1.5 L of 1× TAE at 1.0 V/cm for 48 h at 4°C with buffer circulation.

#### Southern blotting

Southern blotting was performed using a previously described method^57^ unless otherwise indicated, as follows. For DNA transfer, following electrophoresis the gel was soaked in 500 mL of 0.25 M HCl for 30 min, denatured in 500 mL of denaturation solution (1.5 M NaCl, 0.5 M NaOH) for 30 min, and neutralized in 500 mL of neutralization solution (1.5M NaCl, 0.5M Tris, Adjust to pH7.5 with HCl) for 30 min, all at room temperature. The DNA was then capillary-transferred to Hybond-N+ (GE Healthcare) in 10 × SSC overnight. After the transfer, DNA was fixed with 120,000 μJ/cm2 in a Stratalinker (Stratagene, Model 1800), and the membrane was air-dried after washing in milliQ.

To prepare probes, PCR products amplified with HS204/HS205 or HS210/211^51^ were used as templates to generate radioactive probes for detection of ERCs or chromosome XII, respectively. Probes were amplified with the Random Primer DNA Labeling Kit Ver.2 (TaKaRa), and purified by ProbeQuant G-50 Micro Columns (GE Healthcare).

Hybridization was performed using the method described by (Ide S 2010). Briefly, the membrane was incubated in a hybridization bottle with 25 mL of hybridization buffer (10 g/L bovine serum albumin [Nacalai tesque, 01281-84], 0.5 M phosphate buffer [pH 7.2], 70 g/L sodium dodecyl sulfate [SDS], 1 mM EDTA [pH 8.0]) at 65°C for 1 h. The probe was denatured at 100°C, then added, and the membrane was incubated overnight at 65°C. After hybridization, the membrane was washed once for 30 min at 65°C with wash buffer 1 (2 × SSC, 1 g/L SDS), then washed twice for 30 min at 65°C with wash buffer 2 (0.1 × SSC, 1 g/L SDS). The washed membrane was sealed into a plastic film and exposed to a phosphorimaging screen which was scanned on a Typhoon FLA7000 (GE Healthcare). Signal intensities were quantified using FUJIFILM Multi Gauge version 2.0 software (Fujifilm).

#### Flow cytometry analysis

300 μL of saturated yeast culture was inoculated into 3 mL of fresh YPD liquid medium, cultured overnight after dilution and collected the next day. After collection, cells were fixed with 70% ethanol, resuspended in 200 μL of 50 mM sodium citrate (pH 7.4) with 0.25 mg/mL RNaseA and incubated 37°C for 1 h. After RNaseA treatment, 100 μL of 50 mM sodium citrate (pH 7.4) with 0.5 mg/mL Proteinase K (Nacalai) was added, and cells were incubated at 50°C for 1 h. Finally, 300 μL of 50 mM sodium citrate (pH 7.4) with 4 μg/mL propidium iodide (Sigma-Aldrich) was added. Flow cytometry analysis was performed using a BD Accuri C6 Flow Cytometer (BD Bioscience). Signals collected by FL3A channel were plotted.

#### Genomic DNA preparation for Chr.XII sequencing

A saturated culture of each strain was collected and washed once with sterile water. 0.2M Tris-HCl (pH 8.8) mixed with 1/20 volume of β-mercaptoethanol was added to the collected yeast, vortexed well, and this was incubated at 30°C for 30 min. Cells were pelleted, resuspended in a buffer containing 1M Sorbitol, 40 mM Phosphate Buffer with 1 mg/mL Zymolyase 100T (Nacalai), and then incubated at 37°C for 1 h. The cells were then pelleted again, buffer containing 50 mM Tris-HCl (pH8.8), 0.2M NaCl, 0.1M EDTA and 5% SDS was added, followed by incubation at 65°C for 30 min. Equal amounts of phenol/chloroform/isoamyl alcohol (25:24:1) were added and mixed by inversion, and following centrifugation the aqueous layer was transferred to another tube. This step was repeated twice. To the aqueous layer, 1 mL of 100% ethanol was added, the thread-like genomic DNA was transferred into 70% ethanol, and spined down. The precipitate was dissolved in TE (pH 8.0) with 0.08 mg/mL RNase, and this was incubated at 37°C for 1 h. The DNA was again purified with a single phenol/chloroform extraction followed by ethanol precipitation as described above. Finally, the DNA was resuspended in sterile water and used for sequencing.

#### Illumina analysis

The yeast rDNA repeat was amplified by PCR from yeast genomic DNA with oTM302/oTN303. The PCR products were gel-purified and sonicated. Sequencing was performed using Illumina NextSeq with a 66 nt single-end read setting. The sequenced reads were cleaned up using Trim Galore!^54^ and mapped to the yeast reference rDNA sequence derived from sacCer3 S288C strain reference genome using BWA-MEM v0.7.17.^53^ Naive variant caller: https://github.com/blankenberg/nvc was used to identify noncanonical single nucleotide variants in the resulting mapped files.

#### Nanopore analysis

Whole genome sequencing libraries were constructed using yeast genomic DNA following the protocol provided by Oxford Nanopore (SQK-LSK-109). The libraries were sequenced with FLO-FLG001 Flongle flow cells. Basecalling was performed with Guppy v6.4.6. Quality filtering was performed based on read length (>8000 nt) and average Phred quality score (>18). Subsequently, rDNA-derived reads were split into 300 nt bins and mapped to the rDNA reference sequence with BWA-MEM v0.7.17. If the distance between each mapped reads was different from the expected distance by more than 100 nt, the read was considered to contain large structural variation.^34^

### Quantification and statistical analysis

Quantification was performed by using Multi Gauge (FUJIFILM). Statistical significance was calculated using Tukey’s test (Figure 3A; Figure S3) and linear regression model (Figure S1B). Statistical tests were performed in R. Data are presented as mean ± standard error of the mean (SEM). Statistical significance was described as follows: ∗∗∗ = p < 0.001, ∗∗ = p < 0.01, n.s., not significant.
