# Supplementary material for: Replication fork blocking deficiency leads to a reduction of rDNA copy number in budding yeast
Source: iScience. 2024 Feb 6;27(3):109120. doi: 10.1016/j.isci.2024.109120 (PMC10879690; doi:10.1016/j.isci.2024.109120)
Supplement: Document S1. Figures S1–S6 [file mmc1.pdf]

iScience, Volume *27*

Supplemental information

Replication fork blocking deficiency leads to a reduction of rDNA copy number in budding yeast

Taichi Murai, Shuichi Yanagi, Yutaro Hori, and Takehiko Kobayashi

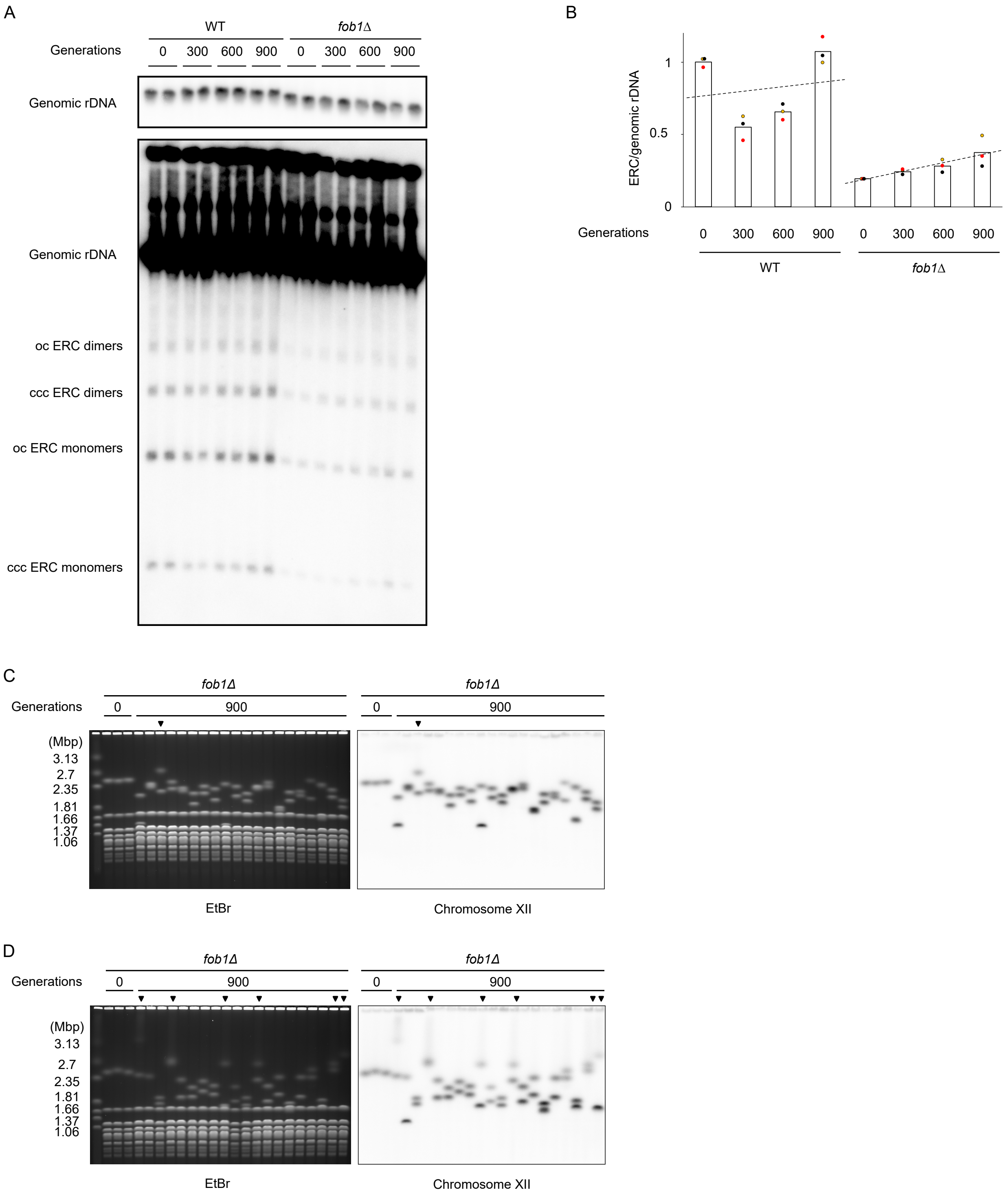

Figure S1, related to Figure 1: ERC accumulation and rDNA copy number in long-cultured cells

(A) DNA from the indicated strains were separated by agarose gel electrophoresis. ERCs were detected by Southern blotting using rDNA specific probes. The top panel is a short exposure to quantify genomic DNA. (B) ERCs indicated in Fig.A were quantified. The sum of indicated ERC signals were divided by genomic rDNA, and each value was normalized by the average of 0-generation wild type. Circles represents each individual data (n=3), and dotted lines indicate a linear regression model for generations in each strain. p values for the models were 0.628 and 0.00207 for wild type and *fob1*Δ, respectively. (C and D) Single colonies were isolated as in Fig.1E for the 0 and 900 generations of *fob1*Δ colony-2 (YTM454 and YTM514) (C) and colony-3 (YTM455 and YTM515) (D). Their genomic DNAs were monitored as in Fig.1C. Arrow heads indicate clones with increased rDNA copies.

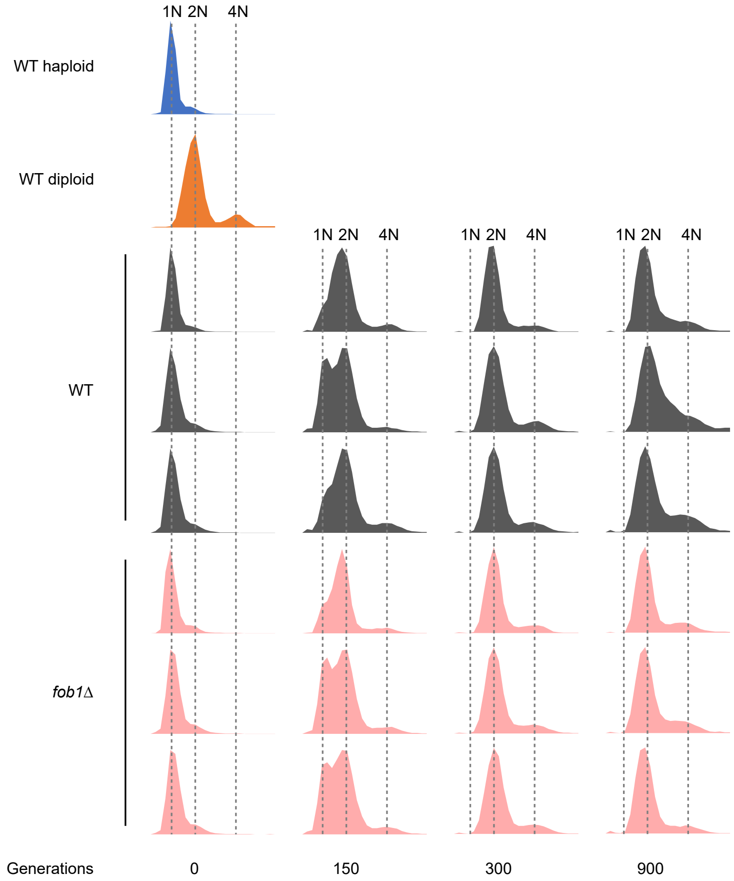

Figure S2, related Figure 1: Flow cytometry of the long-term cultured strains showing diploidization
Long-cultured wild-type (grey) and *fob1Δ* (pink) strains were harvested overnight, stained by propidium iodide (PI) and their fluorescence was plotted as indicated. BY4741 (blue) and BY4743 (orange) were used as haploid and diploid controls, respectively.

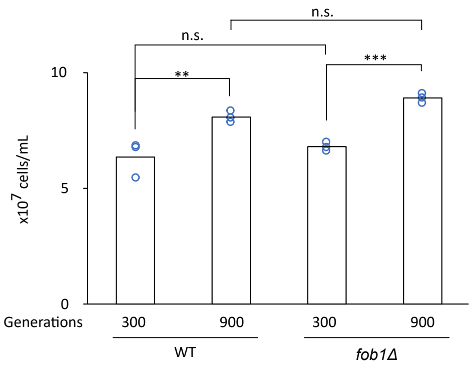

Figure S3, related to Figure 1: Growth rates of long-term-cultured strains
Cells were cultured and their densities measured as in Fig. 3A Bars show the averages of 3 replicates (circles). **: $p < 0.01$, ***: $p < 0.001$, n.s.: $p > 0.05$, Tukey's test.

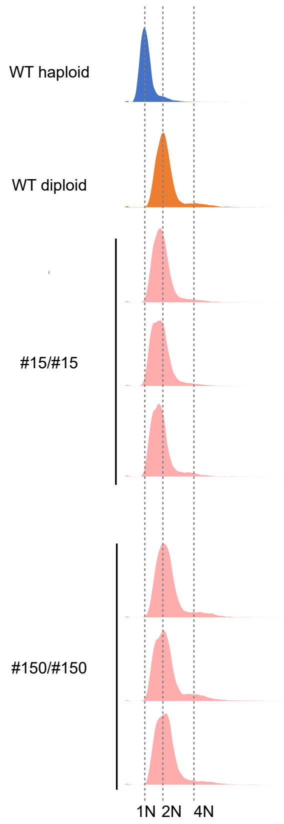

Figure S4, related to Figure 3: Flow cytometry of strains with low copy rDNA
Diploid *fob1*Δ/*fob1*Δ strains with known copy number (#15/#15 or #150/#150) were harvested overnight, stained by propidium iodide (PI) and their fluorescence was plotted as indicated. BY4741 (blue) and BY4743 (orange) were used as haploid and diploid controls, respectively.

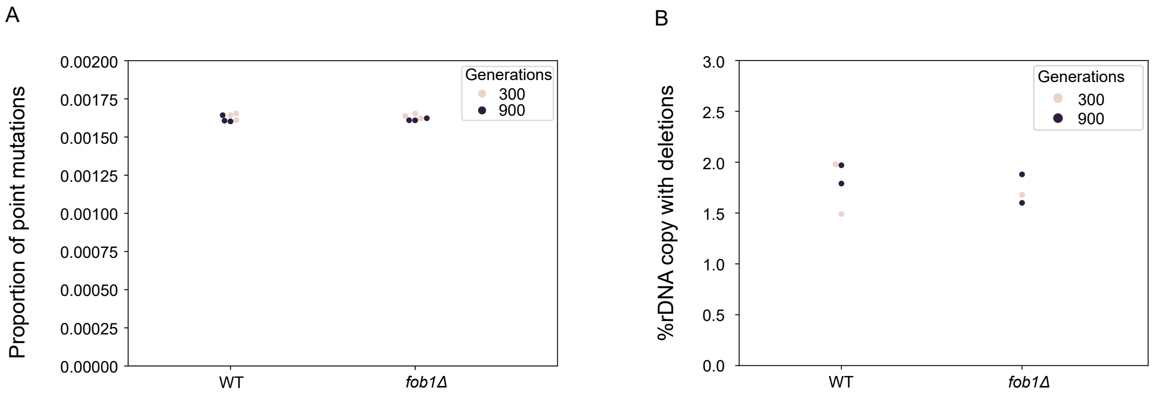

Figure S5, related to Illumina analysis and Nanopore analysis: Comparison of rDNA mutation frequencies between wild-type and *fob1*Δ

(A) The proportion of point mutations from the reference yeast rDNA sequence as determined by Illumina sequences is plotted for wild -type and *fob1*Δ strains after 300 and 900 generations of growth as indicated. To eliminate the influence of inherently variant alleles, we excluded positions where the variant is > 1% in frequency. (B) The proportion of rDNA repeat units harboring deletion mutations > 50 bp in size as determined by Oxford Nanopore sequencing is plotted for wild-type and *fob1*Δ strains after 300 and 900 generations of growth as indicated.

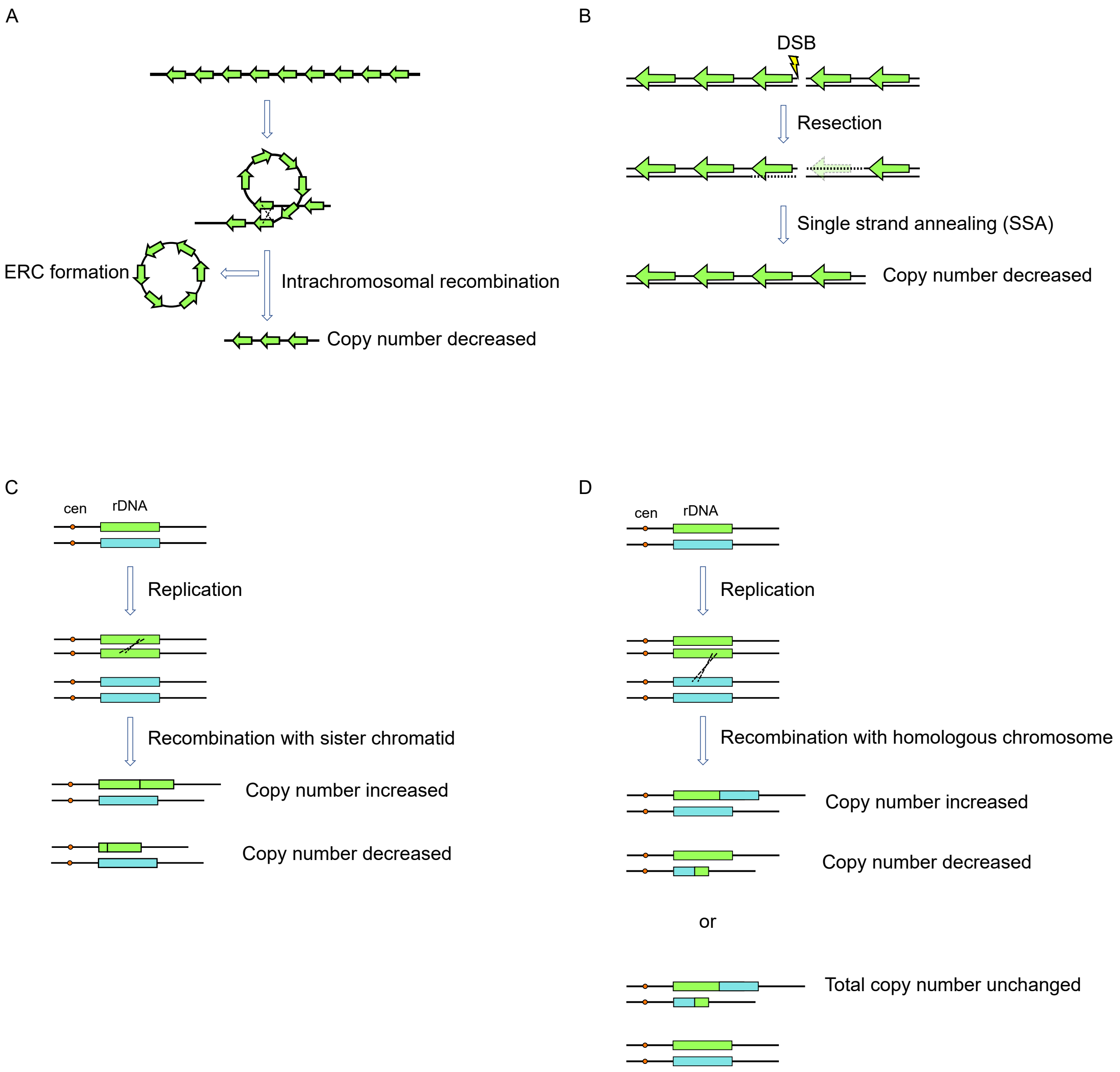

Figure S6, related to Figure 1: Possible models for copy number changes
(A) and (B) rDNA copy number decrease through intrachromosomal recombination (A) and single strand annealing (B). (C) and (D) Possible interchromosomal recombination in diploids. Consequences for recombination with sister chromatids (C) or homologous chromosomes (D) are shown.
